# Sorafenib and everolimus in patients with advanced solid tumors and KRAS‐mutated NSCLC: A phase I trial with early pharmacodynamic FDG‐PET assessment

**DOI:** 10.1002/cam4.3131

**Published:** 2020-05-21

**Authors:** Lucia Nogova, Christian Mattonet, Matthias Scheffler, Max Taubert, Masyar Gardizi, Martin L. Sos, Sebastian Michels, Rieke N. Fischer, Meike Limburg, Diana S.Y. Abdulla, Thorsten Persigehl, Carsten Kobe, Sabine Merkelbach‐Bruse, Jeremy Franklin, Heiko Backes, Roland Schnell, Dirk Behringer, Britta Kaminsky, Martina Eichstaedt, Christoph Stelzer, Martina Kinzig, Fritz Sörgel, Yingying Tian, Lisa Junge, Ahmed A. Suleiman, Sebastian Frechen, Dennis Rokitta, Dongsheng Ouyang, Uwe Fuhr, Reinhard Buettner, Jürgen Wolf

**Affiliations:** ^1^ Department I of Internal Medicine Center for Integrated Oncology Aachen Bonn Cologne Duesseldorf Lung Cancer Group University of Cologne Cologne Germany; ^2^ Onkologische Praxis Moers Moers Germany; ^3^ Faculty of Medicine and University Hospital Cologne Center for Pharmacology Department I of Pharmacology University of Cologne Cologne Germany; ^4^ Faculty of Medicine and University Hospital Cologne Institute for Diagnostics und Intervention Radiology University of Cologne Cologne Germany; ^5^ Faculty of Medicine and University Hospital Cologne Department for Nuclear Medicine University of Cologne Cologne Germany; ^6^ Faculty of Medicine and University Hospital Cologne Institute for Pathology University of Cologne Cologne Germany; ^7^ Faculty of Medicine Institute for Medical Statistics and Bioinformatics University of Cologne Cologne Germany; ^8^ Max Planck Institute for Metabolism Research Cologne Germany; ^9^ Praxis for Medical Oncology and Haematology (PIOH) Frechen Germany; ^10^ Heamatology and Oncology Augusta Hospital Bochum Germany; ^11^ Bethanien Hospital Solingen Germany; ^12^ Medical Oncology and Haematology St. Marien Hospital Düren Germany; ^13^ Institute for Biomedical and Pharmaceutical Research (IBMP) Nürnberg Germany; ^14^ Department of Clinical Pharmacology Xiangya Hospital Central South University Changsha China; ^15^ Hunan Key Laboratory for Bioanalysis of Complex Matrix Samples Changsha China

**Keywords:** FDG‐PET, KRAS mutation, non‐small‐cell lung cancer, pharmacodynamics, pharmacokinetics, Phase‐I trial, solid tumors

## Abstract

**Background:**

Treatment of patients with solid tumors and KRAS mutations remains disappointing. One option is the combined inhibition of pathways involved in RAF‐MEK‐ERK and PI3K‐AKT‐mTOR.

**Methods:**

Patients with relapsed solid tumors were treated with escalating doses of everolimus (E) 2.5‐10.0 mg/d in a 14‐day run‐in phase followed by combination therapy with sorafenib (S) 800 mg/d from day 15. KRAS mutational status was assessed retrospectively in the escalation phase. Extension phase included KRAS‐mutated non–small‐cell lung cancer (NSCLC) only. Pharmacokinetic analyses were accompanied by pharmacodynamics assessment of E by FDG‐PET. Efficacy was assessed by CT scans every 6 weeks of combination.

**Results:**

Of 31 evaluable patients, 15 had KRAS mutation, 4 patients were negative for KRAS mutation, and the KRAS status remained unknown in 12 patients. Dose‐limiting toxicity (DLT) was not reached. The maximum tolerated dose (MTD) was defined as 7.5 mg/d E + 800 mg/d S due to toxicities at previous dose level (10 mg/d E + 800 mg/d S) including leucopenia/thrombopenia III° and pneumonia III° occurring after the DLT interval. The metabolic response rate in FDG‐PET was 17% on day 5 and 20% on day 14. No patient reached partial response in CT scan. Median progression free survival (PFS) and overall survival (OS) were 3.25 and 5.85 months, respectively.

**Conclusions:**

Treatment of patients with relapsed solid tumors with 7.5 mg/d E and 800 mg/d S is safe and feasible. Early metabolic response in FDG‐PET was not confirmed in CT scan several weeks later. The combination of S and E is obviously not sufficient to induce durable responses in patients with KRAS‐mutant solid tumors.

## Graphical abstract

The study investigated the combination of sorafenib and everolimus in patients with solid tumors (dose escalation part) and KRAS mutated NSCLC (expansion part). Although the trial showed manageable safety profile of sorafenib and everolimus, it failed in providing long terms responses for patients with solid tumors and KRAS mutated NSCLC. The novelty of the study was the early pharmacodynamics assessment using FDG‐PET implemented in a phase‐I.

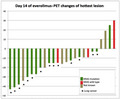

## 1 BACKGROUND

In the majority of advanced solid tumors, chemotherapy and also the newer immune checkpoint inhibitor‐based therapies are of limited efficacy with often modest overall response rates (ORR) and prolongation of overall survival (OS).^1^, ^2^, ^3^ For a subset of cancers, specific inhibition of constitutively activated transforming signal transduction pathways leads to an impressive increase in efficacy in terms of ORR, progression free survival (PFS), and OS.^4^, ^5^, ^6^, ^7^ In particular, in non–small‐cell lung cancer (NSCLC) a substantial number of patients harboring genetic alterations in EGFR, ALK, ROS1, or BRAF can benefit from personalized approved therapies.^5^, ^6^, ^7^, ^8^, ^9^, ^10^, ^11^, ^12^ Currently, further driver alterations such as MET, RET, HER2 are under evaluation in NSCLC.^13^, ^14^, ^15^, ^16^, ^17^

However, one of the longest known oncogenic driver mutations, activated KRAS, has remained untargetable. KRAS mutations occur in several types of solid tumors, most frequently in lung adenocarcinoma (17%), colorectal cancer (19%), and pancreatic cancer (60%).^18^ Loss of the GTPase function triggers downstream signaling of the RAF‐MEK‐ERK‐ and the PI3K‐AKT‐mTOR pathways, both leading to proliferation and angiogenesis.^18^ The inhibition of MEK‐ERK signaling with MEK inhibitors, such as selumetinib or trametinib, showed only modest activity with response rates of about 10%.^19^, ^20^, ^21^ Currently, new small molecule AMG 510 inhibiting KRAS^G12C^ and thus locking it in an inactive state showed very promising results in a phase I study.^22^ However, the small molecule is active just by the G12C mutation in KRAS and seems to work in lung cancer only. Furthermore, long‐term results have to be awaited.

Alternative treatment with immune checkpoints inhibitors provided promising response rates and prolonged PFS and OS in NSCLC as monotherapy in patients with high PD‐L1 expression and in combination with chemotherapy independent of PD‐L1 status.^23^, ^24^, ^25^ Furthermore, patients with colorectal cancer and microsatellite instability benefit from treatment with immune checkpoint inhibitors as well.^26^ However, treatment options for the majority of KRAS mutated patients with advanced solid tumors remains still limited and chemotherapy is still widely used in these patients.

Combined inhibition of the RAS‐RAF‐MEK‐ERK‐ and the PI3K‐AKT‐mTOR pathways might be a conceivable strategy to inhibit KRAS downstream signaling more effectively. Sorafenib is a multikinase inhibitor targeting the extracellular receptors VEGFR/PDGFR/c‐kit (upstream of KRAS) and the intracellular RAF kinase.^27^ It is approved for treatment of metastasized hepatocellular and renal cell carcinoma and for metastatic differentiated thyroid cancer.^28^, ^29^, ^30^ In a small population of 10 patients with NSCLC and KRAS mutation treated with sorafenib monotherapy, two partial responses were observed.^31^

Everolimus is an immunosuppressive drug exerting also an antiproliferative activity by inhibition of mTOR.^32^ Everolimus is approved in renal cell carcinoma, neuroendocrine malignancies, in breast cancer in combination with exemestan, in subependymal giant cell astrocytoma, and in patients with tuberous sclerosis complex.^33^, ^34^, ^35^, ^36^, ^37^ Furthermore, a phase II study showed an overall response rate of 4.7% in unselected NSCLC population.^38^ Previous trials have investigated feasible treatment schedules for the combination of sorafenib and everolimus in various solid malignancies,^39^ renal cell carcinoma,^40^ and hepatocellular carcinoma.^41^ However, patients were not selected according to genetic alterations in these studies.

In NSCLC, it has been shown that molecular imaging with FDG‐ und FLT‐PET can help to detect responses to targeted therapy early and simultaneously enable to identify patients with treatment failure.^42^, ^43^, ^44^, ^45^

Here we present results of a phase I study in patients with solid tumors treated with everolimus and sorafenib. In the dose escalation part, tumor samples were retrospectively analyzed for KRAS mutation. In the expansion phase, only patients with KRAS mutated NSCLC were enrolled. FDG‐PET was used as a pharmacodynamic tool during the run‐in phase with everolimus. As this was a phase I study, the number of patients remained limited.

### 1.1 Patients

Adults with solid tumors were enrolled in the study after failure of standard treatment. In the escalation part, patients without any predefined genetic alteration and for escalation part, patients with NSCLC and KRAS mutations were recruited.

Further key inclusion criteria included measurable disease according to RECIST1.1, performance status ECOG 0‐2, adequate blood count, and normal renal and hepatic functions. Key exclusion criteria comprised any concomitant uncontrolled condition. Patients with brain metastases were excluded, if they required permanent treatment.

In this monocentric study, patients were treated at University Hospital Cologne, Germany.

All patients signed written informed consent before enrolment. The trial was designed and conducted according to the principles of GCP/ICH, the study protocol and the informed consent form were approved by the Ethics Committee at the University Cologne (EudraCT‐number: 2008‐005440‐16).

### 1.2 Molecular diagnostics

No central screening was required for KRAS mutation. KRAS mutation was assessed retrospectively in the dose escalation and prospectively in the dose expansion part. The molecular diagnostics was done either by local pathology or on the central diagnostic platform of the Network Genomic Medicine (NGM) in Cologne. In NGM, KRAS (exons 2 and 3) were analyzed using high resolution melting and positive samples were confirmed by dideoxy (“Sanger”) sequencing as reported previously.^46^ In 2012, next generation sequencing (NGS) was introduced by NGM. Here, KRAS was analyzed in a validated gene panel of 102 amplicons of 14 different genes.^47^

### 1.3 Study design

This phase I study was designed as a monocentric, open‐label one arm trial with the primary objective of determining a safe and tolerable dose for the combination treatment with E and S in patients with advanced solid malignancies (escalation part) and NSCLC patients harboring KRAS mutation (expansion part). Secondary objectives included safety of E and S and their combination, pharmacokinetics (PK), pharmacodynamics (PD) using FDG‐PET, and objective response.

### 1.4 Maximum tolerated dose

The maximum tolerated dose (MTD) of E was determined in a dose‐finding phase by sequential dose escalation on levels 2.5, 5, 7.5, and 10 mg given orally once daily following a 3 + 3 design with predefined criteria for dose‐limiting toxicities (DLT).^48^ Dose‐limiting toxicities was defined as any hematologic toxicity grade 4 as by CTCAE, neutropenia IV°, or thrombocytopenia IV >7 days or any febrile neutropenia IV°, any nonhematologic toxicity IV° or any toxicity requiring hospitalization or requiring interruption of treatment > 14 days occurring within the first 29 days of treatment. After a run‐in period of 14 days with E monotherapy, S was added at a fixed dose of 400 mg orally BID. Combination treatment was continued until progression in CT scan or unacceptable toxicity or patient's withdrawal from any reason before progression. Maximum tolerated dose was defined as the highest dose level where at most 1 of 6 patients experienced DLT. In the extension phase, patients with NSCLC and KRAS mutation were treated with the determined MTD.

### 1.5 FDG‐PET and CT analyses

**Figure 1:**
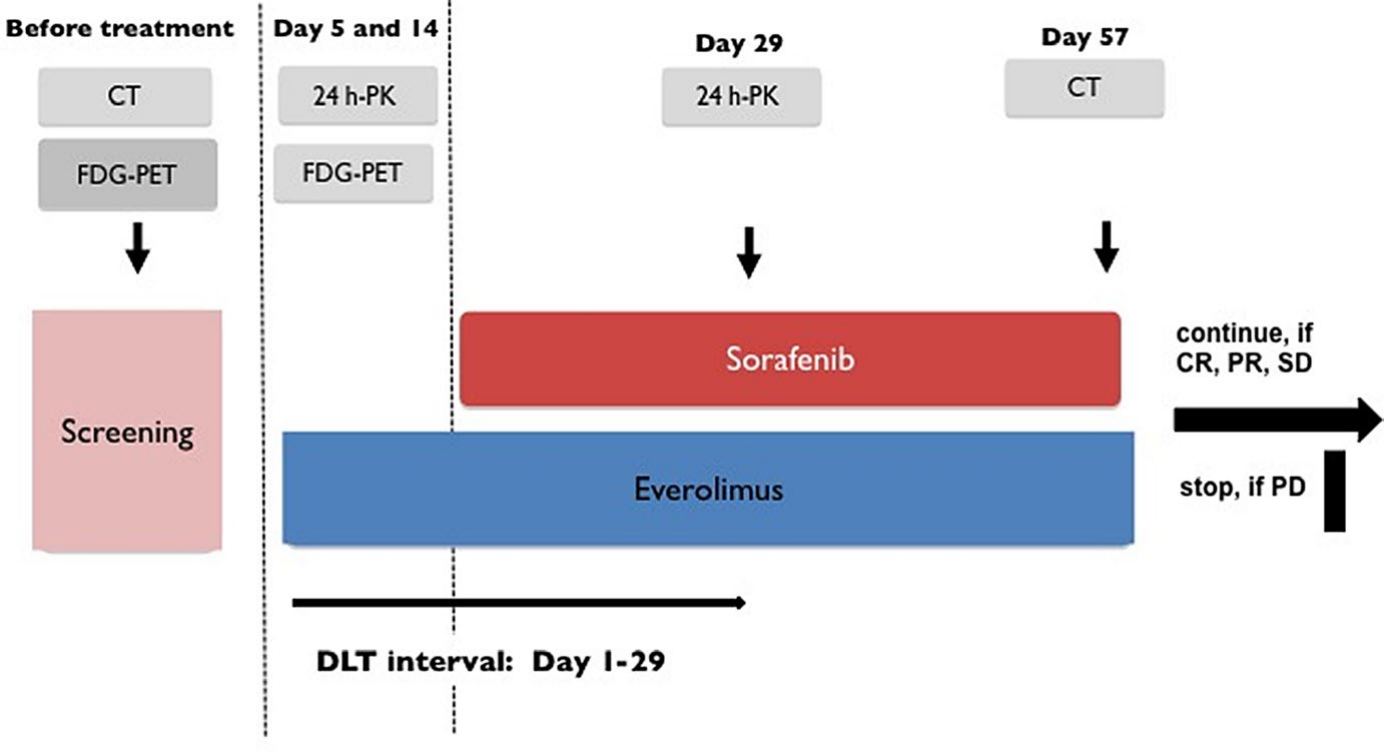
Study flow

In order to assess the predictive value of FDG‐PET, PET scans were performed at baseline and on days 5 and 14 after start of treatment and evaluated according to the PERCIST criteria.^49^ Furthermore, as optional trial procedure, the kinetic rate constants for FDG transport (K1) and FDG metabolism (Ki) were determined at baseline and on days 5 and 14. Objective response was measured with CT scan 8 weeks after start of treatment and then every 6 weeks until progression. All scans were evaluated according to RECIST 1.1.^50^ The treatment flow is summarized in Figure 1.

### 1.6 Pharmacokinetic analyses

Wherever clinically feasible, repeated blood samples for PK analyses were scheduled during a 24 hours period on days 5 (E), 14 (E), and 29 (E + S) of therapy. The PK profile was completed by additional samples at regular study visits. Concentrations of both substances were quantified by liquid chromatography/tandem mass spectrometry (LC‐MS/MS). The lower limits of quantification were 1.052 ng/mL blood (E) and 10.0 ng/mL plasma (S), respectively. Mean precision and absolute accuracy were better than 15% for all analytes. Population pharmacokinetic models were developed for E and S using NONMEM 7.4 (Icon Development Solutions, Ellicott City, MD, USA) and related software^51^, ^52^, ^53^ to describe exposure and potential covariates, including changes during therapy. Empirical Bayes estimates of clearance were obtained for an exploratory evaluation if the respective shrinkage was less than 20%.^54^

### 1.7 Statistical analysis of outcomes

For safety and tolerability analyses, all patients who received any study medication were included (safety set). For efficacy analyses, only patients compliant with the protocol were included (per protocol set). The definition of per protocol set is identical with definition of intention to treat (ITT) set in terms of data analysis.

Maximum tolerated dose was estimated as the highest dose level where ≤ 1 of 6 patients experienced toxicity in terms of DLT. The safety of everolimus and sorafenib was documented using CTCAE version 4.0. Response rates were measured in accordance with RECIST 1.1 criteria. Correlation between KRAS mutation status and response was provided descriptively. Secondary endpoints PFS and OS were analyzed descriptively using the Kaplan‐Meier method. The predictive value of PET response from baseline to days 5 and 14 with respect to PFS and OS was assessed using Cox regression. In an exploratory analysis, the relationships of median individual everolimus and sorafenib clearance and identified covariates for pharmacokinetic parameters were evaluated with respect to PFS and OS using likelihood‐ratio tests. Correlation between kinetic rate constants for FDG transport (K1) and FDG metabolism (Ki) from dynamic FDG PET with SUVs from static FDG‐PET were reported descriptively.

## 2 RESULTS

### 2.1 Characteristics of patients

Thirty‐seven patients with advanced solid malignancies gave informed consent and were screened for eligibility from October 2009 to December 2013. Six of these patients were screening failures. Thirty‐one patients were enrolled and evaluable in terms of the primary objective (ie, included in per‐protocol set), 16 of them suffering from NSCLC (51.6%), and 15 (48.4%) from other malignancies. The mean age of all enrolled patients was 58.5 (41‐77) years with a balanced distribution of male (n = 15, 48.4%) to female patients (n = 16, 51.6%). Most patients with NSCLC were enrolled in the extension phase of the trial (n = 13, 41.9%), all of them with lung adenocarcinoma harboring mutation of KRAS as defined per study protocol. The overall percentage of detected KRAS mutations in all patients (dose finding and extension phase) was 48.4% (15 patients) while the KRAS status was unknown in 35.5% (12 patients). Four patients had wild‐type KRAS. Of 19 patients with known KRAS status, in 18 patients KRAS was tested within the NGM and in 1 patient by local pathology. Of 18 patients tested within the NGM, KRAS was assessed using Sanger sequencing in 17 patients and NGS panel in 1 patient. Most patients presented in fairly good to moderate general condition with a majority of patients showing ECOG 0 (n = 21, 67.7%) (Table 1).

**Table 1:** Patient characteristics (n = 31)

Age	years
Mean	58.48
Range	41‐77
Sex	n (%)
Male	15 (48.4%)
Female	16 (51.6%)
ECOG performance status	n (%)
0	21 (67.7%)
1	8 (25.8%)
2	2 (6.5%)
KRAS status	n (%)
Mutation	15 (48.4%)
Wild type	4 (12.9%)
Unknown	12 (38.7%)
Diagnosis	n (%)
Lung cancer	16 (51.6%)
Adenocarcinoma	15 (48.4%)
Mixed type	1 (3.2%)
Colorectal cancer	3 (9.7%)
Malignant melanoma	3 (9.7%)
Ovarian cancer	2 (6.5%)
Breast cancer	1 (3.2%)
Oesophageal cancer	1 (3.2%)
Pancreatic cancer	1 (3.2%)
Vaginal cancer	1 (3.2%)
Hemagiopericytoma	1 (3.2%)
Leiomyosarcoma	1 (3.2%)
CUP	1 (3.2%)
Previous treatments	median (range)
Surgery	1 (0‐4)
Radiation	1 (0‐4)
Chemotherapy	2.5 (0‐6)
Targeted therapy	0 (0‐2)

### 2.2 Treatment & safety

In the dose‐finding phase, 14 evaluable patients were treated with escalating doses of E (2.5‐10.0 mg/day) and 800 mg/day S; further 17 patients were treated within the extension phase at the determined MTD of 7.5 mg E (dose level III) and 800 mg S. Overall, 20 patients (64.5%) were treated at dose level III (Table 2). Formal criteria of DLT according to the protocol were not met in the dose escalation phase. However, we observed thrombocytopenia requiring dose reduction or interruption of treatment in all patients treated on dose level IV (10 mg everolimus and 800 mg sorafenib) occurring after the DLT interval as defined by protocol (within 29 days after start of treatment). Considering these clinical observations, the Data Safety Monitoring Committee and the sponsor of the trial decided to fix the MTD for the extension phase at dose level III (7.5 mg E and 800 mg S). Treatment‐related toxicities in the escalation and in the expansion are listed in Tables 3 and 4, respectively.

**Table 2:** Dose levels (n = 31)

Dose level	Everolimus (mg/d)	Sorafenib (mg/d)	Number (%) of patients
I	2.5	800	4 (12.9%)
II	5.0	800	3 (9.7%)
III (escalation phase)	7.5	800	3 (9.7%)
III (expansion phase)	7.5	800	17 (54.8%)
IV	10.0	800	4 (12.9%)

**Table 3:** Treatment‐emergent adverse events (TEAEs) in the escalation phase

Everolimus dose (mg/d) TEAEs	2.5 mg		5 mg		7.5 mg		10 mg		All doses
	Gr1/2 (n)	Gr 3/4 (n)	Gr1/2 (n)	Gr3/4 (n)	Gr1/2 (n)	Gr3/4 (n)	Gr1/2 (n)	Gr3/4 (n)	All grades (n)
Hand‐foot‐skin‐reaction	1	1	2		1		3	2	10
Infection	1	1	1	1	4		1	1	10
Rash	4				7		1		8
Mucositis	1	1	4		1		1		8
Diarrhea	3				1		6		7
Skin hypersensitivity	1				2		3		6
Lack of appetite	1				1		3		5
Cough							5		5
Fatigue					1		2		3
Thombocytopenia								3	3
Rhinorrhea	1				1		1		3
Increased GOT				2		1			3
Abdominal pain						1	2		3
Nausea	1				1				2
Hypertension	1						1		2
Nail changes	2								2
Heartburn			2						2
Leucocytopenia				1				1	2
Gastrointestinal ulceration					1		1		2
Sleeplessness							2		2
Dyspnea							1	1	2
Anemia								2	2
Headache							1		2
Muscle pain							2		2
Mouth hypersensitivity	1								1
Epistaxis	1		1						1
Increased GPT						1			1
Increased GGT				1					1
Increased AP				1					1
Neutropenia				1					1
Gastrointestinal bleeding				1					1
Hyperthyreosis					1				1
Constipation						1			1
Pruritus					1				1
Hemorrhoids						1			1
Hematoma					1				1
Alopecia					1				1
Edema							1		1
Dysphagia							1		1
Hoarseness							1		1
Flu like syndrome							1		1
Cardiac ischemia								1	1

**Table 4:** Treatment‐emergent adverse events in the expansion phase

TEAEs	7,5 mg		
	Gr1/2 (n)	Gr 3/4 (n)	All grades
Diarrhea	16		16
Infection	6	2	8
Abdominal pain	5	2	7
Fatigue	7		7
Lack of appetite	7		7
Rash	6		6
Nausea	4		4
Hand‐foot‐skin reaction	3	1	4
Hypersensitive skin	4		4
Mucositis	3		3
Thrombocytopenia	1	3	4
Back pain	3		3
Vomiting	2		3
Cough	4		3
Dyspnea	1	2	3
Sore throat	3		3
Headache	3		3
Anemia	3		3
Bone pain	2		2
Epistaxis	2		2
Colitis	2		2
Pneumonitis	1	1	2
Epistaxis	2		2
Anal fistula		2	2
Dry mouth	1		1
Hemorrhoids	1		1
Alopecia	1		1
Lid edema	1		1
Sleeplessness	1		1
Fever	1		1
Hoarseness	1		1
Hypocalcemia	1		1
Muscle pain	1		1
Chest pain	1		1

### 2.3 Pharmacokinetics

Extensive E blood concentration profiles (up to 9 data points) scheduled for days 5, 14, and 29 of treatment (with up to 1 day deviation) were available for 4, 3, 19, and 4 patients on 2.5, 5, 7.5, and 10 mg daily doses, respectively. S plasma concentration profiles with at least four data points (median 5 data points) on day 29 were obtained for 15 patients. Altogether, 480 data points for E and 122 data points for S were available.

**Figure 2:**
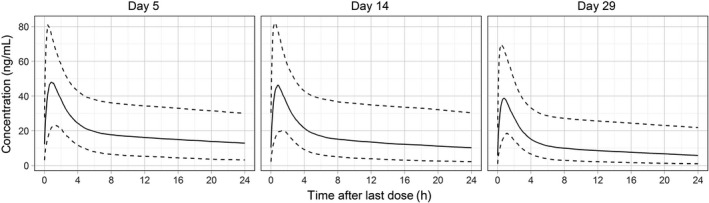
Predicted whole blood concentration vs time profiles of everolimus (point estimates [solid lines] and 90% prediction interval [dashed lines]) for the 7.5 mg QD dose on days 5, 14, and 29 of treatment

A two‐compartment model with linear absorption and elimination was appropriate to describe E blood pharmacokinetics. Bootstrap population estimates (medians and 90% confidence intervals (CIs)) of pharmacokinetic parameters were absorption constant 0.74 (0.58‐1.27) h^−1^, central and peripheral volumes of distribution 46.6 (29.9‐100.1) L and 612 (488‐790) L, intercompartmental clearance 63 (53‐72) L/h, and initial systemic clearance 15.4 (11.7‐19.2) L/h, respectively. Shrinkage of E systemic clearance was 5%. Exposure to E increased essentially linearly with the dose. Interestingly, E concentrations decreased during the course of treatment. To account these changes, data points were sorted into three bins (days 0‐10, days 11‐20, and after day 21), and interoccasion variability was tested for PK parameters. E clearance for the second and third period increased by 23 (10‐48) % and 81 (53‐125)%, respectively, compared to the first period (Figure 2). Among the covariate relationships tested, PK of everolimus was not influenced by age, body weight, hematocrit, and sex, but clearance decreased from 127% to 82% of the population estimate for lowest (0.2 mg/dL) to highest (0.7 mg/dL) observed plasma bilirubin concentrations, explaining 17% of residual interindividual variability in clearance. Individual empirical Bayes estimates were used to calculate exposure for one 12 hour dosing interval in terms of C_max,τ_ and AUC_τ_ (Table 5).

**Table 5:** Everolimus exposure within one dosing interval of approximately 24 h according to dose and to treatment period (results of individual post‐hoc estimates based on the population pharmacokinetic model)

Parameter	Day of everolimus treatment	2.5 mg QD	5 mg QD	7.5 mg QD	10 mg QD
AUC_τ_ (h* ng/mL blood)	Days 1‐10 (scheduled: day 5)	(n = 4) 115 (1.48)	(n = 3) 284 (1.46)	(n = 19) 451 (1.79)	(n = 3) 302 (1.59)
	Days 11‐20 (scheduled: day 14)	(n = 3) 121 (1.71)	(n = 2) 152 (1.05)	(n = 6) 241 (1.37)	(n = 4) 349 (1.88)
	After Day 29 (scheduled: day 29, with sorafenib)	(n = 3) 69 (1.68)	(n = 2) 137 (1.12)	(n = 6) 287 (1.91)	(n = 3) 240 (1.36)

Values are given as geometric mean (coefficient of variation). Numbers of patients (n) vary because not for all patients samples were available in all periods.

For S, the limited number of available data points precluded the development of an independent pharmacokinetic model. To provide an estimate of exposure, structure and parameter estimates from a published model^55^ were used. Interindividual variability of oral bioavailability (IIV_F_) and the fraction of cleared S subject to enterohepatic circulation (*F*_ent_) were estimated from S plasma concentrations of this study for all patients with at least four data points on day 29. Volumes of distribution and clearances were scaled allometrically with body weight (standardized to 70 kg, exponent 1 for volumes, 0.75 for clearances), which allowed a reasonable fit of the model to the observed concentrations. Shrinkage of S systemic clearance was 12%. The estimated *F*_ent_ was 0.58 and bioavailability varied with a coefficient of variation of 49%. Exposure in terms of AUC_τ_ values for the dosing interval studied (day 29, actual mean ± SD duration of interval 11.8 ± 0.5 hours) based on individual empirical Bayes estimates was (geometric mean) 131 (CV 56%) μg*h/mL in subjects receiving only doses of S 400 mg BID until day 29. Three subjects received at least one dose of 200 mg just before day 29 (mean ± SD of sorafenib doses: 395 ± 32 mg), in these subjects AUC_τ_ values were 85 μg*h/mL (CV 28%).

### 2.4 Efficacy

On day 5, PET was performed in 30 patients. In one patient, new metabolic active lesions were detected as marker of progressive disease. Five patients reached partial metabolic response (PMR), of those four patients had KRAS mutation. Twenty‐three patients showed stable metabolic disease (SMD) and one patient had progressive metabolic disease (PMD).

**Figure 3:**
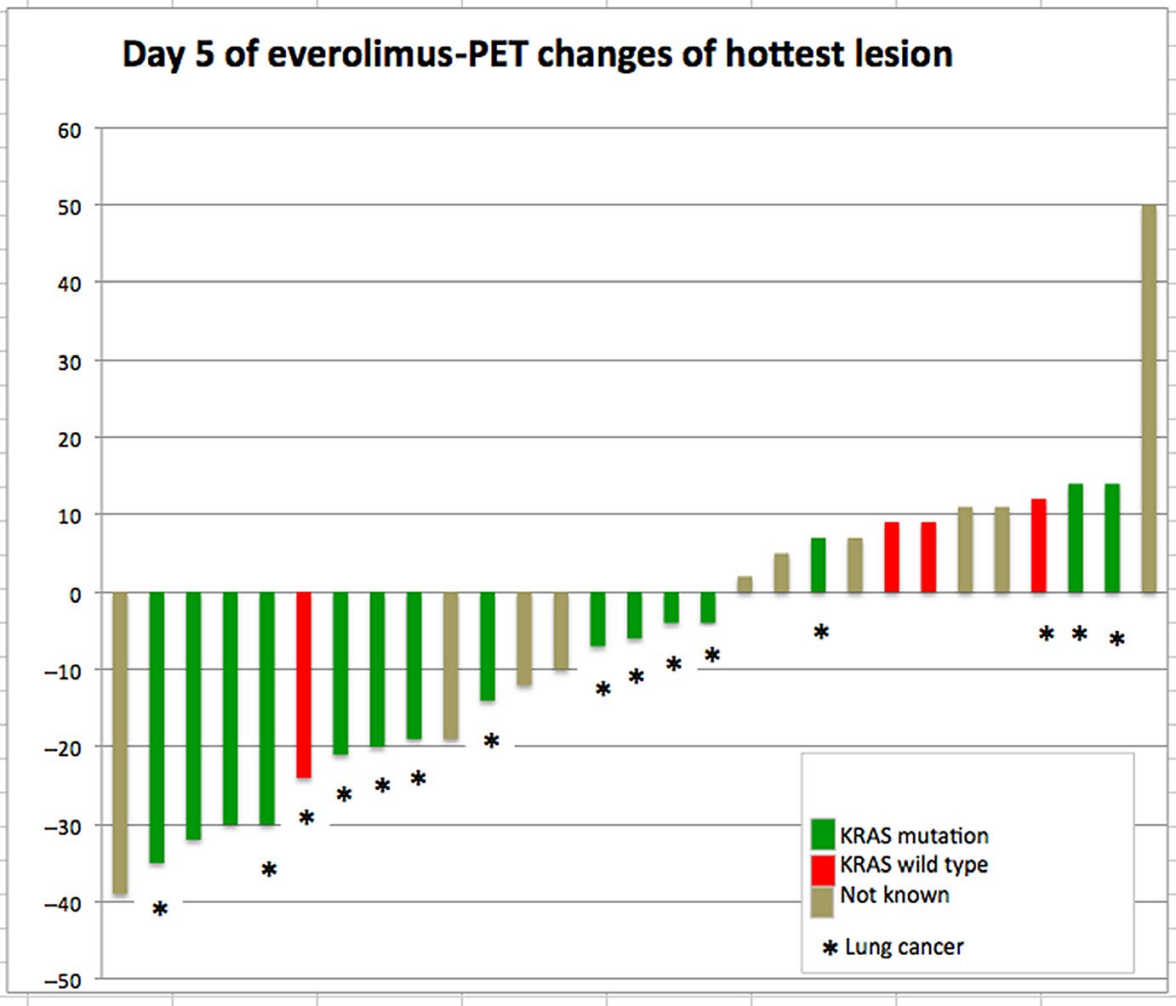
PET changes on day 5 of everolimus. Treatment response to everolimus on day 5 in FDG‐PET shown as changes of FDG hottest lesion according to PERCIST

**Figure 4:**
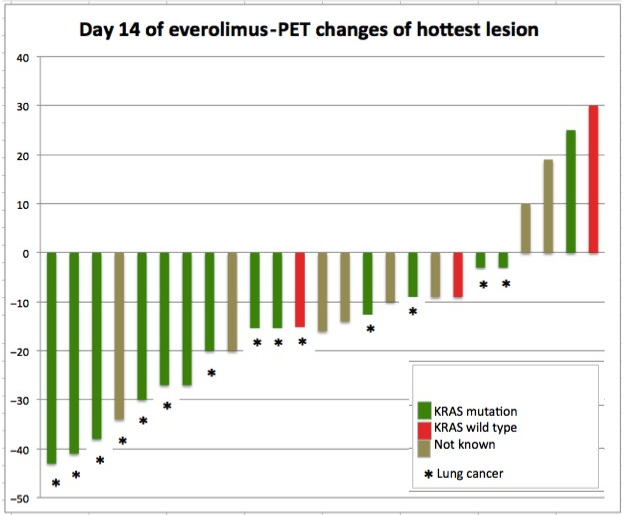
PET changes on day 14 of everolimus. Treatment response to everolimus on day 14 in FDG‐PET shown as changes of FDG hottest lesion according to PERCIST

**Figure A1:**
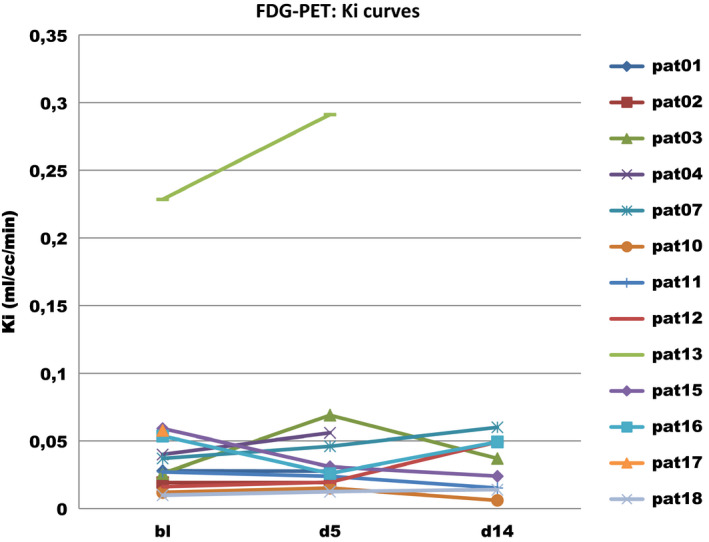
Ki values measured on baseline, day 5 and 14 in patients treated with everolimus

**Figure A2:**
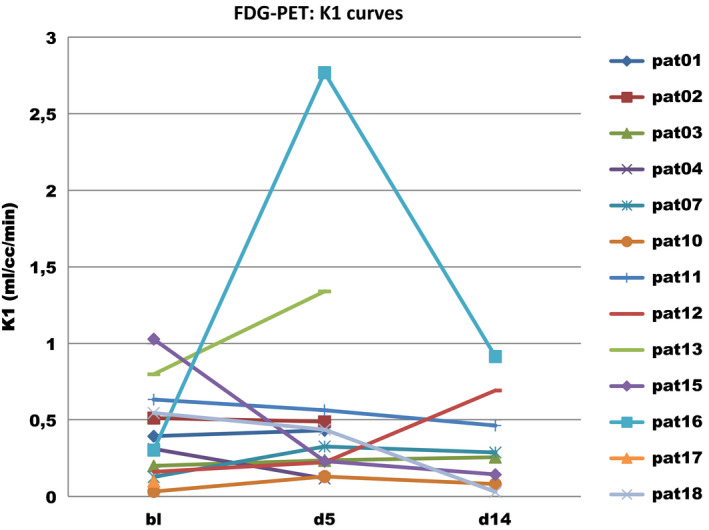
K1 values measured on baseline, day 5 and 14 in patients treated with everolimus

On day 14, 25 patients received PET, 5 of them showed PMR: 4 with KRAS mutation and 1 with unknown KRAS mutational status. One patient presented with PMD and 19 patients showed SMD (Figures 3 and 4). No differences in K1 and Ki values were seen between baseline, days 5 and 14 (Figures A1 and A2).

A more pronounced reduction in PET metrics from baseline to day 14 was linked to a significant decrease in hazard of progression (hazard ratio [95% CI] for a unit‐decrease in PET response of 0.82 [0.68‐0.97]) and death (0.79 [0.64‐0.97]). However, this depended on two influential patients with KRAS‐mutated NSCLC with the most distinct observed decreases in PET response (−9.4 and −8.3, PET metric at day 14 minus baseline). When these two patients were excluded, no significant link of PET response and OS/PFS remained.

Higher concentrations of baseline bilirubin were significantly related to a higher hazard of death (15 [1.3‐160]), but not of progression (*P* = .16). Median individual E clearance was significantly related to PFS (0.97 [0.95‐1.00]) while no significant relationship was observed for OS (*P* = .85). Sorafenib clearance was neither significantly related to PFS (*P* = .08) nor OS (*P* = .66).

Six patients died before the first CT restaging at week 8, 5 of them due to progression of the underlying disease. One patient with NSCLC died on day 39 after the start of treatment from sudden cardiac arrest. This event was assessed as not related to the study medication. Four patients had progression before first CT restaging.

CT scan at week 8 was performed in 21 patients, none of them experienced complete or partial response as defined by RECIST 1.1. Twenty patients showed SD. Of these 20 patients, 11 patients showed moderate reduction in sum of longest diameter of all tumor manifestations with a best change of −22.2% in a patient with NSCLC and KRAS mutation. One patient showed progressive disease at week 8.

**Figure 5:**
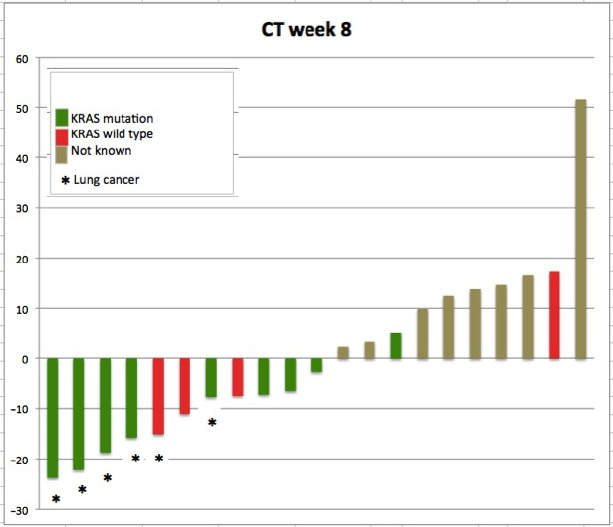
CT changes on week 8 (day 56) of treatment. Response to combination of everolimus and sorafenib in CT at week 8 of study treatment showing changes according to RECIST 1.1

**Figure 6:**
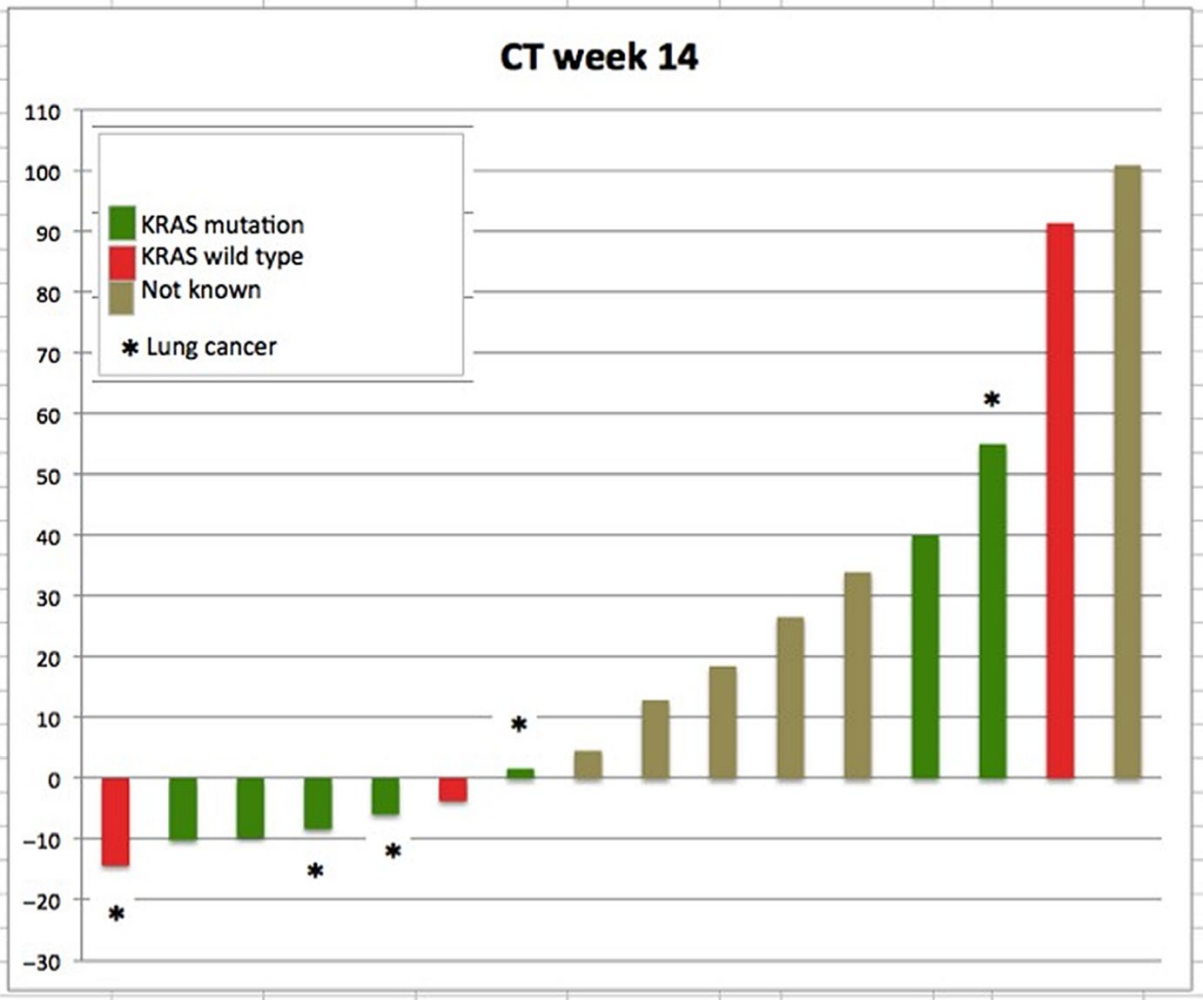
CT changes on week 14 of treatment. Response to combination of everolimus and sorafenib in CT at week 14 of study treatment showing changes according to RECIST 1.1

Concerning the CT at week 14, 4 patients were progressive before the second CT restaging and 1 patient refused CT due to worsening condition. Finally, CT was performed in 15 patients at week 14. In 10 patients (38.1%) the SD was confirmed, 5 patients showed PD (Figures 5 and 6). No patient reached response (CR/PR) in subsequent CT scans.

Five patients (16.1%) discontinued treatment without evidence of progression: three patients due to adverse events (1 patient with bronchial infection of grade III, 1 patient with hand‐foot syndrome grade II, and 1 patient with epistaxis of grade II, all events related to study medication), 1 patient due to individual decision, and 1 patient due to newly diagnosed concomitant colorectal cancer (investigator decision).

**Figure 7:**
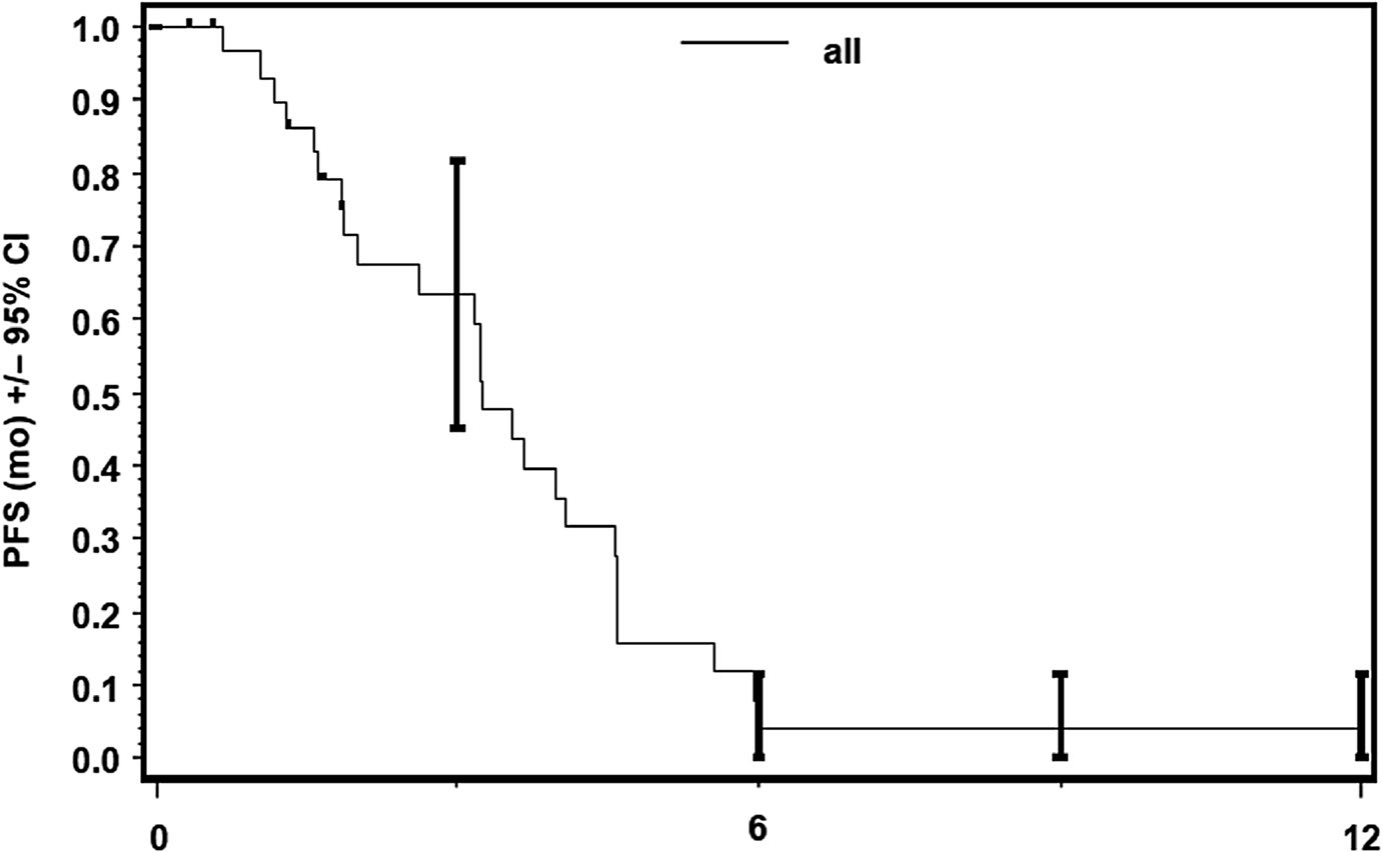
Progression‐free survival (PFS), ITT. Progression‐free survival in ITT (per protocol) population with median PFS of 3.25 mo

**Figure 8:**
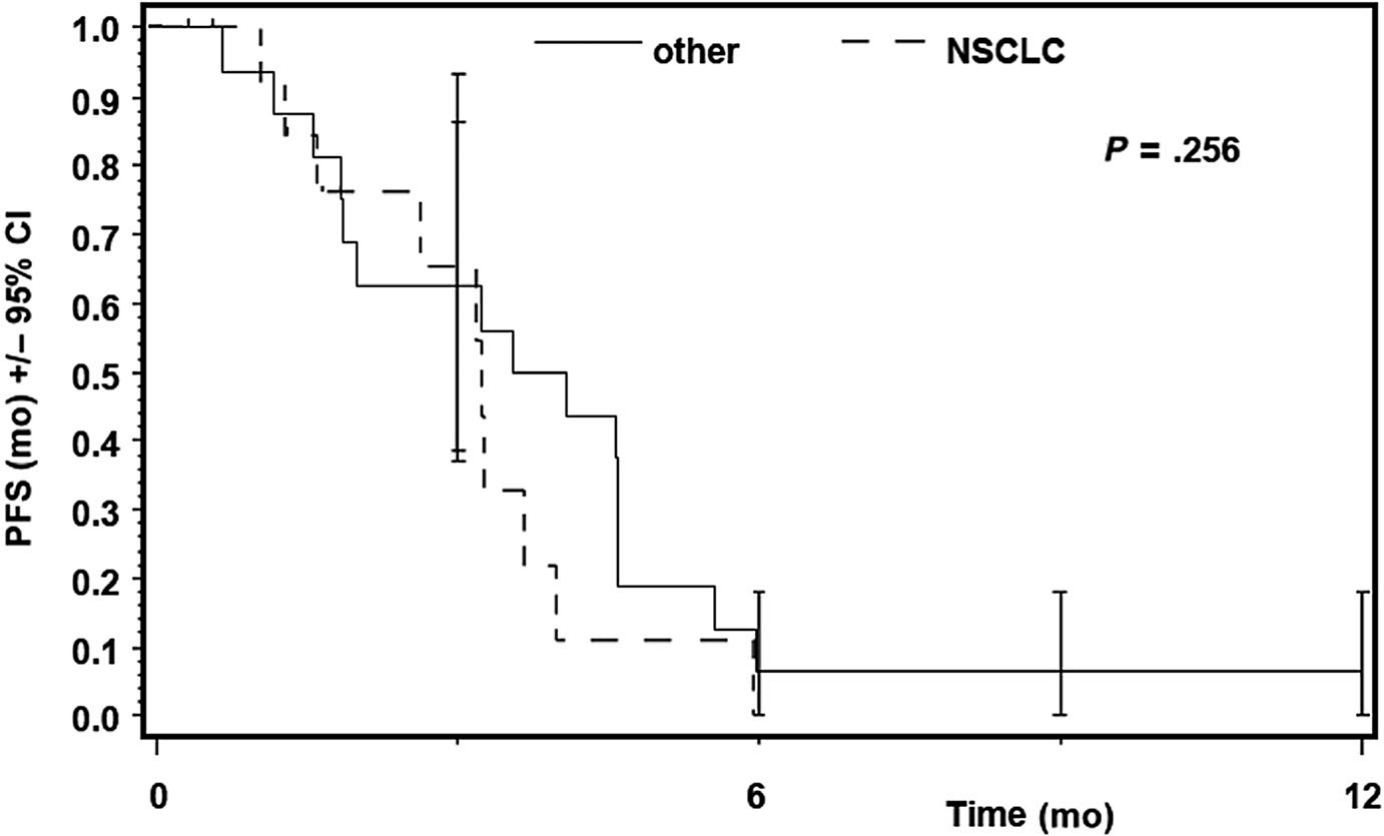
Progression‐free survival in patients with solid tumours and NSCLC. The statistical analysis showed no significant differences between the two groups

Thirty‐one patients were included in ITT population, 26 patients had progressive disease at time point of data cutoff. Progression‐free survival of 26 patients ranged from 32 to 497 days after start of treatment in Kaplan‐Meier analysis. Median PFS was 3.25 month (95% CI, 2.00‐4.07) (Figure 7). There was no statistically significant difference in median PFS between patients with NSCLC and other solid tumors (*P* = .26; Figure 8).

**Figure 9:**
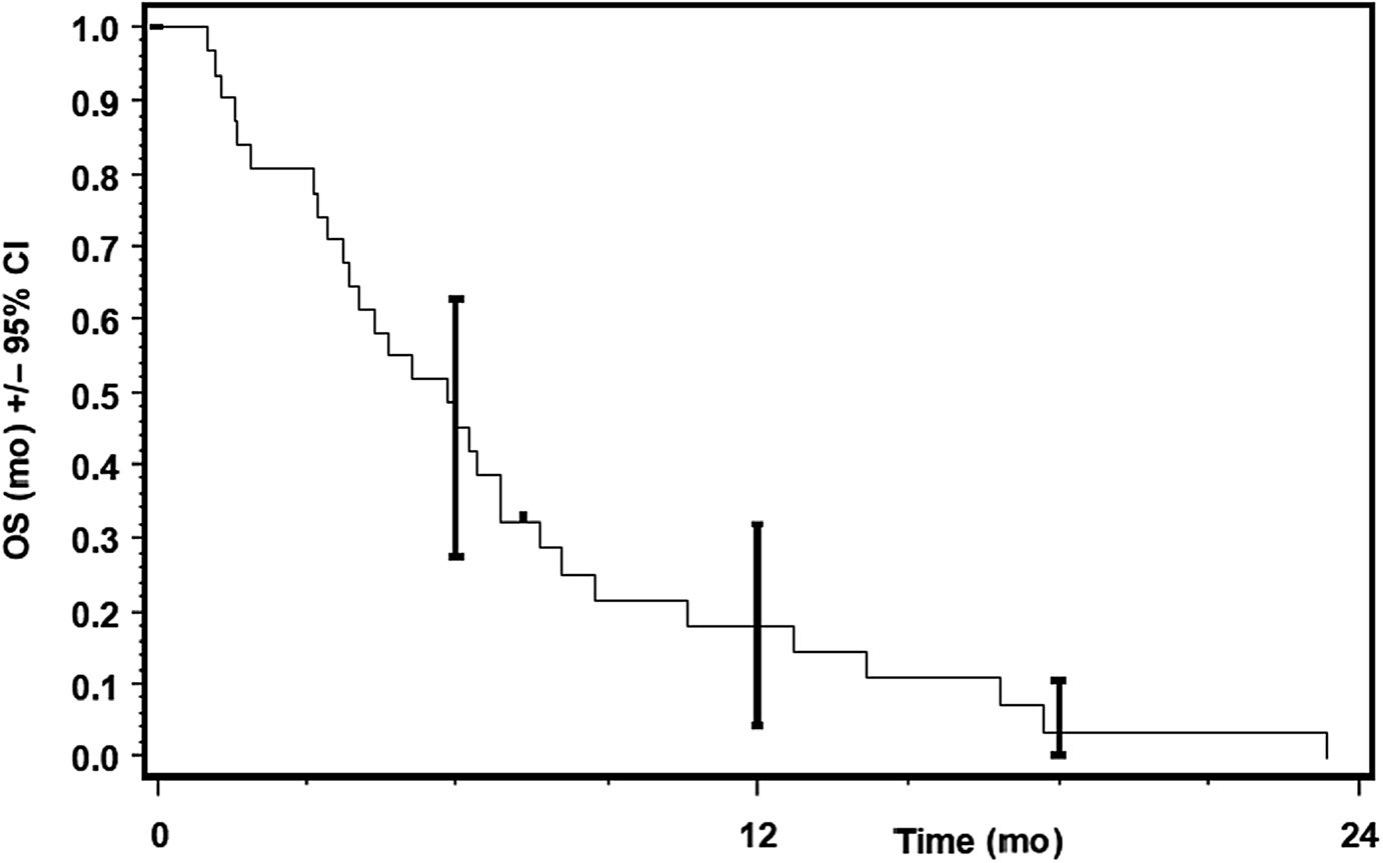
Overall survival (OS), ITT. Overall survival in ITT (per protocol) population with median survival of 5.85 mo

**Figure 10:**
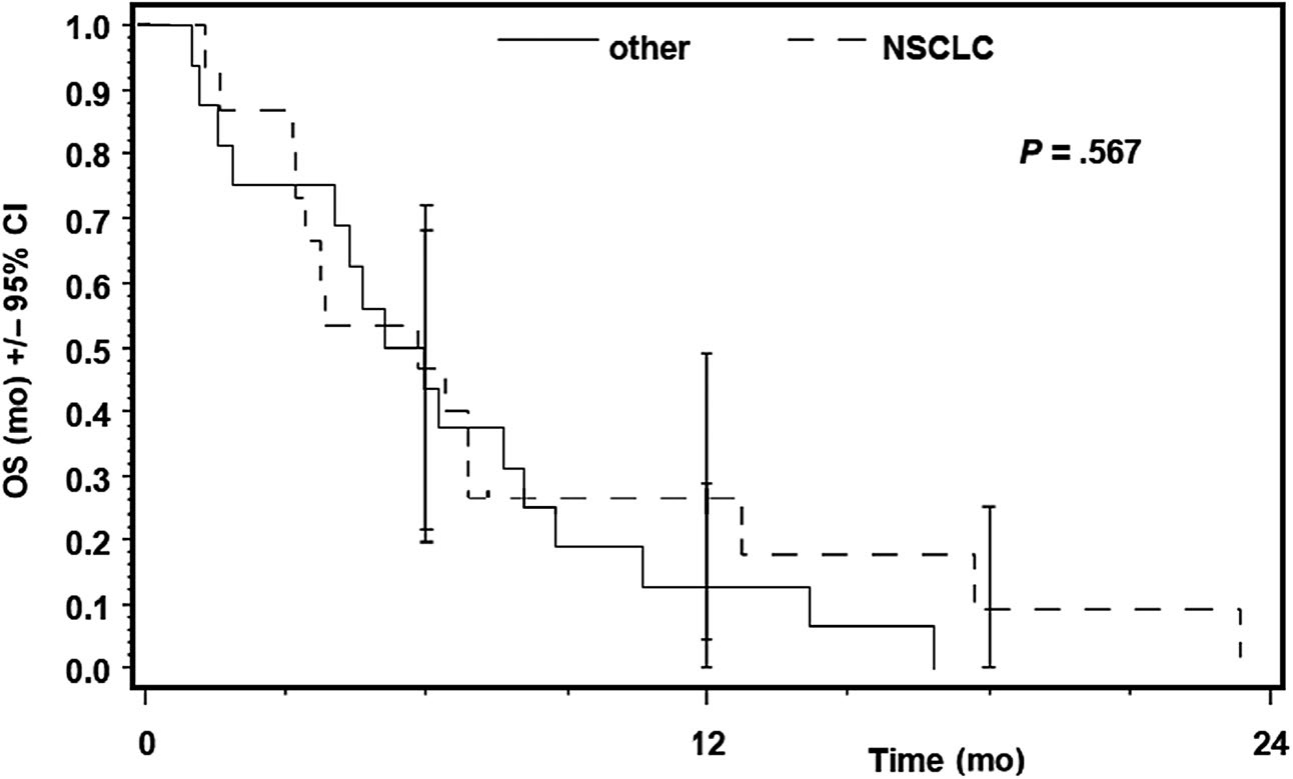
Overall survival (OS), ITT vs NSCLC. Overall survival in patients with solid tumours and NSCLC. The statistical analysis showed no significant differences between the two groups

One patient with neuroendocrine tumor of unknown origin stayed on treatment for 513 days. At time of analysis, 30 patients from ITT population died. All patients died between 32 and 538 days after start of treatment. Median overall survival according to Kaplan‐Meier analysis was 5.85 months (95% CI, 3.75‐6.9) (Figure 9). There was no statistically significant difference between patients with NSCLC and other solid tumors (*P* = .57) (Figure 10).

## 3 DISCUSSION

The results of this trial describe a safe and feasible treatment schedule with 7.5 mg E and 800 mg S for patients with advanced solid malignancies.

The MTD established in our trial is higher than published before: with 35 mg/week everolimus + 400 mg/day sorafenib,^40^ 2.5 mg/day everolimus + 600 mg/day sorafenib^39^ or 2.5 mg/day everolimus + 800 mg/day sorafenib.^41^ One potential explanation for this effect might be the sequential start of treatment with E monotherapy for 2 weeks followed by combination treatment of E and S, possibly helping patients better tolerate the treatment related adverse events.

Furthermore, we observed slightly different toxicities than previously published. The dose limiting toxicities in the previous combinations trials were hand‐foot syndrome and asthenia.^39^, ^40^, ^41^ Although formal DLT criteria were not met in our trial, thrombocytopenia turned out to be the most common dose limiting side effect occurring after more than 30 days of treatment (ie, after 15 days of combination treatment) consistent with published data in patients with hepatocellular carcinoma (HCC).^41^

Exposure to both E and S was similar to other reports.^55^, ^56^, ^57^ The quantitative assessment of individual exposure may be helpful to understand both adverse and desired effects of treatment. The mechanism for the increasing clearance of E with duration of treatment remains unclear. E exposure based on whole‐blood concentrations was reported to be lower in patients with lower hematocrit values,^58^ but hematocrit was not a significant covariate in the present study. It is tempting to speculate that early improvement in overall health conditions along with the therapy might also have improved drug biotransformation, either by improving hepatic function^59^ or by a reduction of circulating cytokines.^60^ These changes may indicate that tolerability studies conducted at the beginning of treatment may not reflect tolerability including MTD for prolonged treatment.

This might also explain the observed relationship between PFS and everolimus clearance. Since an increase in everolimus clearance causes a decreased drug exposure, the observed increase in PFS seems contradictory. However, the observed increase in PFS might result from improved overall health and thus increased biotransformation, suggesting that reduced exposure is of subordinate importance. This is supported by the relationship between OS and baseline bilirubin, which suggests that improved liver function outweighs the potentially disadvantageous effects on pharmacokinetic in terms of a more rapid drug elimination. However, this evaluation was only exploratory since it was based on a limited selection of pharmacokinetic parameters. For example, time‐varying PK and the variability in volumes of distribution were not considered. The complex interactions between PK of everolimus and sorafenib and overall health require further evaluations, including a more extended PK/PD modeling.

In terms of efficacy of treatment, the imaging procedures demonstrated heterogeneous findings. Although the PET response rates in terms of PMR on days 5 and 14 were 17% and 20%, respectively, no patient experienced objective partial response as defined by RECIST 1.1 in CT scan later. Furthermore, no correlation between PMR and PFS or/and OS was found in Cox regression analysis.

The early PET results in our study are not consistent with data published previously, indicating a good predictive value of early PET scan for further treatment efficacy with erlotinib or everolimus in patients with NSCLC.^42^, ^43^, ^44^, ^45^ However, by analyzing PET responses with regard to KRAS mutation, we saw four patients with KRAS mutation among five PMRs (1/5 patients had unknown KRAS status) on day 5 and 14.

This may underline recently published preclinical and translational data demonstrating that in many cases KRAS mutation represents a subclonal mutation occurring with several other oncogenic drivers.^61^, ^62^, ^63^ It would explain an early response by inhibition of the PIK3CA/AKT/mTOR pathway involved in RAS signaling. However, CT scans with mild tumor growth or stable disease at weeks 8 and 14 could indicate that this effect was probably overcome by oncogenic signaling of co‐mutations or bypassing the RAS up‐ and/or downstream. Although this hypothesis remains to be confirmed and we did not screen archived tumor biopsies for further mutations (except of one case with NGS testing, which showed no co‐occurring mutations), the trial findings are in line with published preclinical and clinical findings that inhibition of RAS/RAF/AKT signaling only might be insufficient in treatment of KRAS mutated patients.^19^, ^20^

It should be noted that individual patients have experienced surprisingly long periods of stable disease in our trial, 1 patient with neuroendocrine carcinoma of unknown origin with the longest time on treatment of 17.1 months and fairly good quality of life. This seems to be consistent with previously published data showing the efficacy of E in treatment of patients with advanced pancreatic NEC.^64^

However, based on low number of patients in this study, the results are limited. The low number of patients is mainly caused due to the design of a phase I study. So far, only 17 patients were treated on maximum tolerated dose and only 13 patients had KRAS mutated lung cancer. This number is surely too low to answer the question if the combination of S and E is sufficient to induce responses in KRAS mutated NSCLC. However, as we saw almost no durable tumor shrinkage in these patients, we concluded that the combination is possibly not enough effective in these patients. Overall, this study demonstrates a safe and feasible schedule for the combination treatment with E and S at a higher dose (7.5mg+800mg) than previously published. In general, the treatment with 800mg S and 7.5mg E was well tolerated. The observed increase of E clearance during treatment suggests that tolerability limits may need to be reassessed according to the duration of treatment. Although everolimus treatment showed early metabolic responses, these responses could not be transferred in later CT findings. Thus, our study did not significantly improve treatment of KRAS‐mutated patients with solid tumors by using the combination of E and S. Our results also question the role of early FDG‐PET for predicting CT‐confirmed objective responses or even a longer PFS and OS, at least in this patient population. To improve treatment of patients with KRAS mutation, new drugs or drug combinations should be evaluated in clinical trials with thoroughly conducted translational analyses to understand the biological mechanism underling response and treatment failure.

## CONFLICTS OF INTEREST

LN has received honoraria/travel expenses and/or had an advisory role by Pfizer, Celgene, Novartis, Bayer, Roche, Boehringer Ingelheim, Janssen, BMS, and Takeda. MS has had an advisory role and/or paid talks by Boehringer Ingelheim, Mediolanum Biosciences, Novartis, Roche, and Takeda. He has received a scientific support from AMGEN and travel support from Boehringer Ingelheim, Mediolanum Biosciences, and BMS. SMB has received grant from Novartis and personal fees from BMS, Novartis, Pfizer, Roche Pharma, and AstraZeneca. AAS is a current employee of AbbVie Deutschland GmbH, and is a holder of AbbVie stock. RNF has had an advisory role by BMS and Roche and has received Honoraria from BMS, MSD, Roche, Boehringer Ingelheim, and AstraZeneca. JW has had an advisory role, received honoraria and travel fees from Abbvie, AstraZeneca, BMS, Boehringer‐Ingelheim, Chugai, Ignyta, Lilly MSD, Novartis, Pfizer, and Roche. DSYA has had an advisory role and received lecture fees from BMS, Boehringer‐Ingelheim, MSD, Novartis, Roche, Healthcare Consulting Cologne, Abbvie. DSYA has received travel funding from AstraZeneca, BMS, Boehringer‐Ingelheim, MSD, Novartis, Roche, and Abbvie. LJ worked during the study conduction at Institute for Pharmacology at University Cologne. Currently she works at IQWiG in Germany (Institut für Qualität und Wirtschaflichkeit im Gesundheitswesen). The institution of LN, MS, RNF, ML, SM, DSYA, and JW has received a research funding from Novartis, Pfizer, BMS, MSD, Roche, Boehringer Ingelheim, Bayer, and Janssen.

## AUTHORS CONTRIBUTION

Authors contributed to the study and the article as follows: Conceptualization: LN, CM, MS, MLS, JW, MG, CK, JF, HB, CS, and UF. Data curation: LN, CM, MS, MTa, TP, CK, JF, HB, FS, UF, and JW. Formal analysis: LN, CM, MTa, TP, CK, SMB, JF, HB, CS, FS, DO, UF, RB, and JW. Funding acquisition: JW and LN. Investigation: LN, CM, MS, MTa, MG, MLS, SM, RNF, DSYA, TP, CK, SMB, HB, RS, DB, BK, ME, CS, YT, LJ, AAS, SF, DR, DO, UF, RB, and JW. Methodology: LN, CM, MS, JF, JW, MLS, TP, CK, SMB, JF, HB, CS, FS, UF, and RB. Project administration: MTh, LN, CM, MS, and MG. Resources: UF, JF, MTa, TP, SMB, JF, CS, FS, JW, and RB. Software: UF, JF, MTa, TP, SMB, JF, HB, CS, FS, UF, vRB. Supervision: LN, JW, UF, and RB. Validation: LN, JW, UF, CM, MS, MTa, MG, TP, JF, HB, CS, FS, UF, and RB. Visualization: LN, MS, JF, UF, HB, CS, and FS. Writing—original draft: LN, JW, UF, MS, CM, RNF, and JF. Writing—review and editing: LN and MS.

## PRESENTATIONS

The design of the trial and partial results were presented as posters at ASCO annual meetings between 2012 and 2017.

## Data Availability

The data that support the findings of this study are available from the corresponding author upon request.
